# The Biological Factor in Infant Feeding

**Published:** 1909-04-24

**Authors:** 


### Diseases of Children.

### THE BIOLOGICAL FACTOR IN INFANT FEEDING.

The laws of animal life are just as applicable to the infant as to the young of other species. Each develops as the result of the fertilisation of a single cell, and in the early stages of existence the embryos of the higher animals are closely alike; specialisation is a later process. Similarly the young, after birth, normally live on the maternal milk until they are able to digest the ordinary diet of the adult of the species. Yet the adult diet may be herbivorous, carnivorous, or omnivorous. Either the maternal milk is of such a nature that it will develop the digestive organs in a special manner suitable for dealing with the future diet, or there is a biological potentiality at the basis of development which leads to the specialisation of function quite independently of the nature of the diet. Obviously the latter is the true explanation, for no amount of cow's milk fed to an infant will modify its stomach into any resemblance to that of the ox.

The elementary constituents of diet are the same in all animals, whether young or full-grown, but the biological need is that these elements are supplied in suitable forms and proportions. Oats and hay are natural foods for a horse, but just as unsuitable for man as meat and puddings would be for a horse. Even different species require similar foods in different forms or quantities. Grass and hay are digested in much larger quantities by the bovine than the equine species, because of the configuration of the stomach. There is a biological difference in the nutritive powers of the young during the stage of milk feeding. Were it not for this the milk of all animals might be used indiscriminately, leaving to the individual the choice of selecting what is requisite and discarding the remainder. To a great extent this is true, for with care an infant can be trained to digest pure cow's milk. Moreover, we frequently come across infants who have been fed on various diets, often apparently irrational, and yet have developed quite satisfactorily. Such a result is not surprising, for the divergence in character of the milk of different animals during the suckling period is not very great. And, as far as is known, the young of all animals secrete digestive juices which contain similar ferments and exert a similar chemical action on the food. Their digestive organs are practically identical at birth, though they develop very differently afterwards.

The maternal milk clots or curdles in the stomach in a manner suitable for assisting the development of the stomach. The woman, mare, and ass secrete a milk poor in casein, and the clot is fine and not coherent. Cow's milk forms a tough resistant curd which stimulates the motor powers of the stomach.

In the search for a substitute for cow's milk too much attention has been devoted to the chemistry of milk, and the problem has been regarded as mainly chemical, the biological basis being quite ignored. Comparisons are made between the results of various methods of feeding in which the diet, though a modified cow's milk, is totally different from human milk. Milk is a living nutritive fluid with many biochemical properties. Take colostrum, for instance, and realise its importance. It is especially rich in antigens, bodies which stimulate the tissues to the production of specific antibodies of the class of agglutins and precipitins. According to Langer, there are no antigens in the blood of the new-born calf, but they appear in a few hours after suckling. They are very scanty in the blood of the calf if, instead of being allowed to suckle its dam, it is suckled by another cow which has calved a week or two previously. Recent experiments have further demonstrated the importance of maternal nursing during the first few days of life. Moro found that of guinea pigs artificially fed from birth 80 per cent. died, whereas if they were suckled for one day 40 per cent., and if for two or three days only 10 per cent. died under smilar conditions.

Another important point is the presence of ferments, such as katalase and antibacterial alexins, and some antiscorbutic substance in fresh milk. These substances are readily destroyed by heat; and the staler the milk the weaker is it in biochemical properties. Chemically we make use of many varieties of casein in the various modification of cow's milk. The proteins consist of casein and soluble albumens, and there is a certain amount of support for the view that the albumens are merely nutritive, whereas the casein is essential for development. Casein is sometimes called caseinogen, calcium casein, or bicaseinate of calcium. With rennin it forms calcium paracasein, a soft curd which readily passes through the pylorus. In early infancy there is little or no hydrochloric acid secreted. Later, the acid formed acts on the calcium paracasein and converts it into a tougher free paracasein, which is acted on by pepsin. Casein forms with lime water, calcium caseinate; with bicarbonate of soda, calcium sodium caseinate; with citrate of soda, sodium caseinate and calcium citrate; with hydrochloric acid, casein hydrochloride. All these compounds are unacted on by rennin, and consequently their effect is to enable the milk to pass through the stomach without curdling. The digestion is then entirely intestinal. In these respects the milk can be manipulated so as to make it acceptable to the infant's stomach. The results again prove that infants, like other organisms, possess the power of adapting themselves to their food supply. It remains obvious that no modification of cow's milk can be regarded as identical with human milk, no matter how accurately the percentages of the different constituents is devised, for it is deprived of its bio-chemical properties. Maternal nursing remains unapproachable. But the biological factors of life enable the young to appropriate what is good and reject that which is evil in many apparently unsuitable diets.

